# 4 Survey and clinical implications on the isolation of mycobacteria in the waterline of dental units

**DOI:** 10.1017/ash.2026.10461

**Published:** 2026-06-23

**Authors:** Sara Keller, Jessica Fernandez, Mikaela Harmsen, Kiran Correa, Fiona Grady, Pranita Tamma, Susan Henderson, Alison Laffan, Tasnuva Liu, Roy Ahn, Carly Parry, Prashila Dullabh

**Affiliations:** 1 Johns Hopkins University; 2 NORC at the University of Chicago; 3 Johns Hopkins; 4 Ahrq; 5 Johns Hopkins University, Armstrong Institute for Patient Safety and Quality; 6 NORC

## Abstract

**Background:** Antibiotic-resistant infections cause 2 million illnesses and 35,000 deaths annually in the U.S., driven partly by unnecessary antibiotic prescribing. Emerging evidence raises concern over prescription rates in primary and urgent care telemedicine settings, where limitations to conducting physical exams and fewer dedicated antibiotic stewardship resources pose challenges. More information is needed about how to implement antibiotic stewardship in these settings. To reduce inappropriate antibiotic prescribing over telemedicine, we implemented the first large-scale quality improvement (QI) intervention for antibiotic stewardship in telemedicine. **Methods:** The Safety Program for Telemedicine enrolled 522 practices representing 49 organizations offering telemedicine in an 18-month QI program. Participating organizations varied in size (1-100+ clinicians), care type (e.g., primary, urgent, pediatric), and structure (e.g., direct-to-consumer, integrated health system). The program included: 1) 18 webinars on antibiotic stewardship, diagnosing and treating common infections over telemedicine, virtual physical exams, and patient communication; 2) QI adviser support; 3) office hours; 4) practical tools (e.g., patient and clinician handouts); 5) benchmarking reports; and 6) sustainability guidance. We conducted 25 virtual interviews with program participants from 23 practices to identify challenges and opportunities to improve antibiotic prescribing in telemedicine and program facilitators and barriers. Transcripts were coded in NVivo. Using rapid deductive qualitative analysis, we organized data into predefined domains while identifying emergent themes and synthesized summaries to identify cross-cutting patterns. Monthly QI adviser reports on implementation successes and challenges were also qualitatively analyzed to explore themes. **Results:** Key themes revealed 1) challenges to appropriate antibiotic prescribing in telemedicine, 2) resources to support antibiotic stewardship, 3) facilitators to successful program implementation, and 4) barriers to program engagement. Challenges reported by participants included concerns about patient satisfaction, limitations in conducting physical exams, and inadequate telemedicine-specific education. Patient communication tools and telemedicine-specific clinical guidance were noted as resources to support appropriate prescribing. Facilitators to successful program implementation included live and asynchronous learning options, case-based materials in various formats (discussion guides, handouts, slides), one-on-one support, and sharing prescribing data in benchmarking reports. Barriers included limited provider time, competing organizational priorities, and difficulties extracting data from electronic health record systems. **Conclusions:** Leaders of antibiotic stewardship efforts in telemedicine settings can apply these findings by offering telemedicine-specific guidance to clinicians, flexible learning, ongoing support/coaching, individualized benchmarking reports, and proactively addressing patient satisfaction through patient education. This program also provides a scalable model for implementing QI interventions in telemedicine settings that can be adapted to other clinical areas.

